# The rising toll of dengue cases in Pakistan every year: An incipient crisis

**DOI:** 10.1016/j.amsu.2022.103549

**Published:** 2022-03-31

**Authors:** Ubaid Khan, Saleha Azeem

**Affiliations:** Department of Medicine, King Edward Medical University Lahore, Pakistan

Dear Editor

The primary aim of this letter is to throw light on the rising cases of dengue infection, its transmission, and death tolls in Pakistan and to discuss the dynamic steps that Pakistan's government has taken to combat this infection. Dengue is primarily transmitted through the bite of infected “*Aedes aegypti”* and “*Aedes albopictus”* that belongs to the family “*Flaviridae*.” The signs and symptoms of dengue fever start appearing within seven days after the bite of the mosquito. The symptoms of dengue include pyrexia, flu, pain in the eyes, circulatory shock syndrome, dengue hemorrhagic fever, thrombocytopenia, and low heme-concentration [1] (see Fig. 1, Fig. 2).

**Fig. 1:**
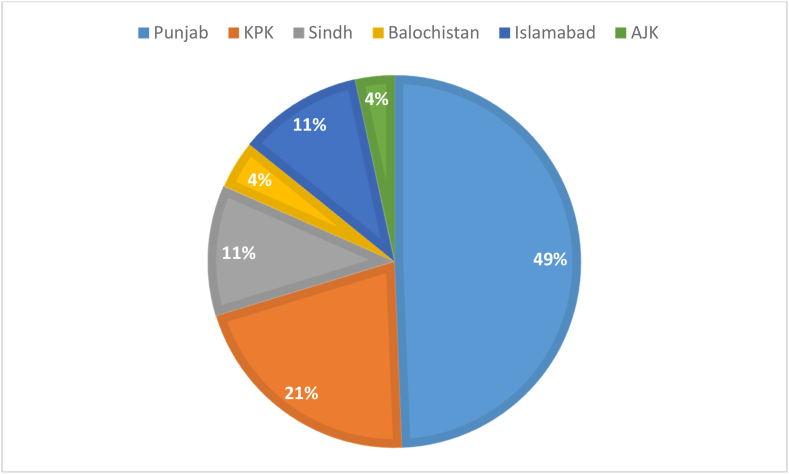
Cases of dengue infection across the country.

**Fig. 2:**
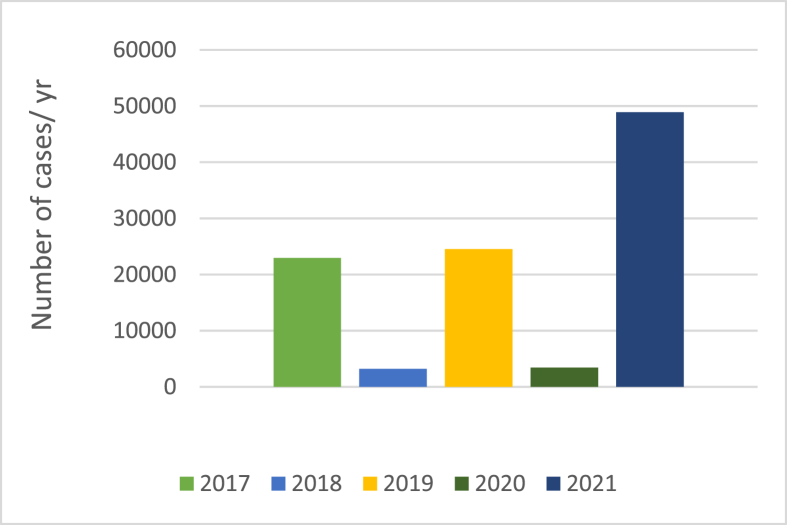
Cases of dengue infection in Pakistan over the years.

World Health Organization (WHO) reported that dengue had targeted mainly humans in South Asia. The healthcare sector of Pakistan has reported its lethal effects due to unhealthy food, edibles with improper sanitation among the population [2].

As COVID-19 infection has been claiming lives daily in Pakistan, medical experts have warned another looming threat of dengue infection rising across the country and some areas have been declared as “sensitive” by health authorities due to dengue larva found active in the water. Another report published by Gulf news highlighted the rising dengue cases in KPK, Punjab, and Islamabad. If well-coordinated efforts are not considered, COVID-19 and dengue could wreck circumstances across the country [3].

For the year 2021, according to World Health Organization (WHO), 48,906 cases including 183 deaths have been reported in Pakistan, and the situation is highly alarming due to rising cases. According to DAWN news, more people are testing positive for dengue fever daily all over the country. As of November 25, 2021, Punjab had reported 24,146 cases since January with Lahore being affected the most. Similarly, Khyber Pakhtunkhwa reported 10,223 cases, Sindh reported 5,548 cases, federally-administered ICT reported 5,261 cases, Balochistan reported 2054 cases, and AJK reported 1674 cases[4]. If immediate preventive measures are not taken situation will be worse, especially in Punjab.

According to the report of District Health Officer (DHO) Dr. Zaeem Zia, due to severe illness around 35 patients had been admitted to “Federal Services Government Polyclinic Hospital and the Pakistan Institute of Medical Sciences (PIMS) by October 2021[5].”

In KPK, the number of cases also seems to be on the rise. KPK health statistics indicated during the survey 2021, many areas of Peshawar city have been declared dengue sensitive, including Shaheen Muslim Town, Tehkal, Landi Arbab, due to the presence of dengue larvae in water. The Focal Person *“Integrated Vector Management Program (IVMP)”* Dr. Khalid told the Express Tribune about the 1st case of dengue. He said it was detected on July 14, 2021, from LandiKotal Tehsil.

Moreover, he said, we are receiving 2 or 3 cases per day. Now, it is reported that Sultan Khel is the central area where Dengue fever is spreading quickly among the population. Government team members have arranged sprays in some areas of Landikotal, but our resources are limited to perform door-to-door spray, he further added [6]. According to news reports, two fatalities have also been recorded in Manshera city of KPK in October 2021. Overall, 10,223 cases have been documented from January to November 2021 in KPK[7]

Furthermore, local media reported that three people died in Karachi due to dengue in October. Since the beginning of October 1255 cases have been detected in Karachi due to weather changes and rains. In the latest report, health officials demonstrated that 7808 cases have been reported across Sindh [8].

According to the National Institute of Health (NIH), Islamabad, the cases reported in Pakistan in 2017, 2018, 2019, and 2020 were 22938, more than 3200, 24547, and 3442 respectively. By November 2021, 48906 cases had been reported for that year [9]. In the period between November 19, 2021, and December 10, 2021, 16388 cases were reported. In a similar period in 2020, only 1153 cases were reported [10]. Almost every year or every alternate year, Pakistan faces a dengue epidemic with increasing severity and mortality despite all the efforts of the government. Without such efforts, the epidemic would be much worse.

According to government officials, targeted operations have been conducted, such as “*eliminating mosquito larvae”* to eradicate mosquito larvae in different cities of KPK and Punjab. Repellents and barriers are standard methods to protect from mosquito bites and vector control [11]. The government of Pakistan has advised people to remove standing water pools from near their homes and residencies where mosquitoes breed. The “*anti-dengue day”* has also been documented to inform and educate people about dengue fever in the past years. The Punjab Chief Minister Usman Buzdar issued strict instructions for mobilizing field teams and surveillance for public and private places.

Furthermore, the KPK government started an anti-dengue campaign in which approximately 15,000 houses have been sprayed. Recently, *“KPK Information Technology Board (ITB)”* has launched a mobile application to eliminate dengue. The main advantage of this app is that people can identify the symptoms of dengue, which helps create awareness among people. These initiatives will help people to combat this lethal virus [12].

## Ethical approval

N/A.

## Sources of funding

There is no source of funding.

## Consent

N/A.

## Author contribution

All authors have contributed equally.

## Registration of research studies

N/A.

## Guarantor

Ubaid khan is guarantor.

## Declaration of competing interest

All authors declare that there is no source of funding.
